# Laparoscopic Lateral Suspension (LLS) for Pelvic Organ Prolapse (POP): Update and Systematic Review of Prospective and Randomised Trials

**DOI:** 10.3390/jcm14093056

**Published:** 2025-04-29

**Authors:** Francesco Plotti, Arianna Martinelli, Corrado Terranova, Carlo De Cicco Nardone, Roberto Montera, Daniela Luvero, Federica Guzzo, Violante Di Donato, Gianna Barbara Cundari, Serena Manco, Roberto Angioli

**Affiliations:** 1 Research Unit of Gynaecology, Department of Medicine and Surgery, Università Campus Bio-Medico di Roma, 00128 Rome, Italy; 2 Fondazione Policlinico Universitario Campus Bio-Medico, Via Alvaro del Portillo 200, 00128 Rome, Italy; 3 Department of Gynecological, Obstetrical and Urological Sciences, “Sapienza” University of Rome, 00185 Rome, Italy

**Keywords:** urogynecologic surgery, pelvic organ prolapse, laparoscopic lateral suspension

## Abstract

**Background**: Pelvic organ prolapse (POP) significantly impacts women’s quality of life, especially in postmenopausal patients. Although laparoscopic sacrocolpopexy (LSC) is the gold standard for advanced apical prolapse, its complexity and risk of complications have led to alternative approaches like laparoscopic lateral suspension (LLS), a minimally invasive technique with promising results. **Methods**: A comprehensive search using PubMed databases was performed. The search was conducted from June 2024 to September 2024. The search string used was as follows: (pelvic organ prolapse) AND (lateral suspension) OR (laparoscopic lateral suspension). We included randomized controlled trials, prospective cohort studies, prospective observational studies, and case studies. We excluded retrospective studies, small case series, case reports, and articles not published in English. All selected articles were screened based on the titles and abstracts. Relevant data were extracted and tabulated. **Results**: An overall number of 12 studies were included in our analysis. LLS demonstrated high anatomical success rates: 91.15% for the anterior, 94.95% for the central, and 86.55% for the posterior compartments. The randomized controlled studies exhibit comparable effectiveness between both methods (LLS vs. LSC) and LLS appears to be the best option for anterior repair or anterior–apical repair. Patient satisfaction rates exceeded 90%, with reduced operative times (123 ± 33 min and 193 ± 55.6 min for ALS and ASC, respectively). According to the Claiven–Dindo scale, 0.17% of postoperative complications were graded more than III. The rate of mesh erosion was 0% to 10%. The technique showed particular benefit for uterine preservation and in obese patients but was less effective for severe posterior prolapse. **Conclusions**: Laparoscopic lateral suspension offers a safe, effective alternative for POP management, with significant anatomical and functional benefits. Its minimally invasive nature, shorter surgery time, and high satisfaction rates make it suitable for tailored patient care. Further studies should standardize evaluation metrics and assess long-term outcomes. The review was not registered. No funding was received. The authors declare no competing interests.

## 1. Introduction

Pelvic organ prolapse (POP) is a prevalent condition that can significantly impair a woman’s quality of life. It refers to the downward displacement of one or more vaginal or uterine segments, including the anterior or posterior vaginal walls, the cervix, or the vaginal apex, which may involve the vault or surgical scar following hysterectomy. The symptoms a woman may encounter could be vaginal pressure and bulge, as well as voiding, defecatory, and sexual dysfunction, all of which can negatively impact their quality of life [1]. Its diagnosis is often misunderstood. The prevalence of POP is reported to be 3–6% when diagnosed based on the presence of symptoms but it rises to 41–50% when assessed through gynecological examination [2]. By the age of 80, the lifetime risk of undergoing at least one surgical procedure for urinary incontinence or POP is approximately 11.1% [3].

Women with severe POP frequently have multi-compartmental prolapse, and the suspension of the central prolapse is a crucial part of a long-lasting surgical fix for women with severe prolapse [4]. Surgical intervention for POP is generally recommended only for symptomatic women. While various surgical techniques are available, there are currently no definitive guidelines to determine the optimal approach. A recent Cochrane review published in 2023 demonstrated the superior outcomes of sacrocolpopexy compared to vaginal surgical techniques among the available options [5]. Currently, laparoscopic sacrocolpopexy using synthetic mesh is regarded as a safe and effective procedure [6]. This technique is widely considered the gold standard for POP repair. However, it requires extensive dissection and fixation of the prosthesis to the sacral promontory, which can be technically demanding and carries risks of neurovascular complications or ureteral injuries [7]. In the past two decades, novel minimally invasive surgical approaches have gained increasing attention in gynecological surgery. One such approach, laparoscopic lateral suspension (LLS)—as introduced by Dubuisson et al.—offers a refined technique for addressing POP and related conditions, demonstrating promising outcomes [8]. Unlike sacrocolpopexy, the LLS technique does not require dissection at the sacrum or sacral promontory, thereby reducing the risk of severe complications. Using a synthetic T-shaped mesh, LLS can be performed with or without concomitant hysterectomy, making it particularly suitable for women wishing to preserve their uterus [9]. Over the past decade, numerous studies have been published on LLS, showing comparable objective cure rates to those of sacrohysteropexy and sacrocolpopexy [10]. However, despite the growing body of literature on laparoscopic lateral suspension (LLS), the available evidence remains fragmented and heterogeneous, and no systematic review has yet comprehensively evaluated its anatomical, functional, and safety outcomes. This review provides a comprehensive analysis of LLS focusing on anatomical and functional outcomes, as well as intraoperative and postoperative complications associated with the procedure.

## 2. Materials and Methods

**Figure 1:**
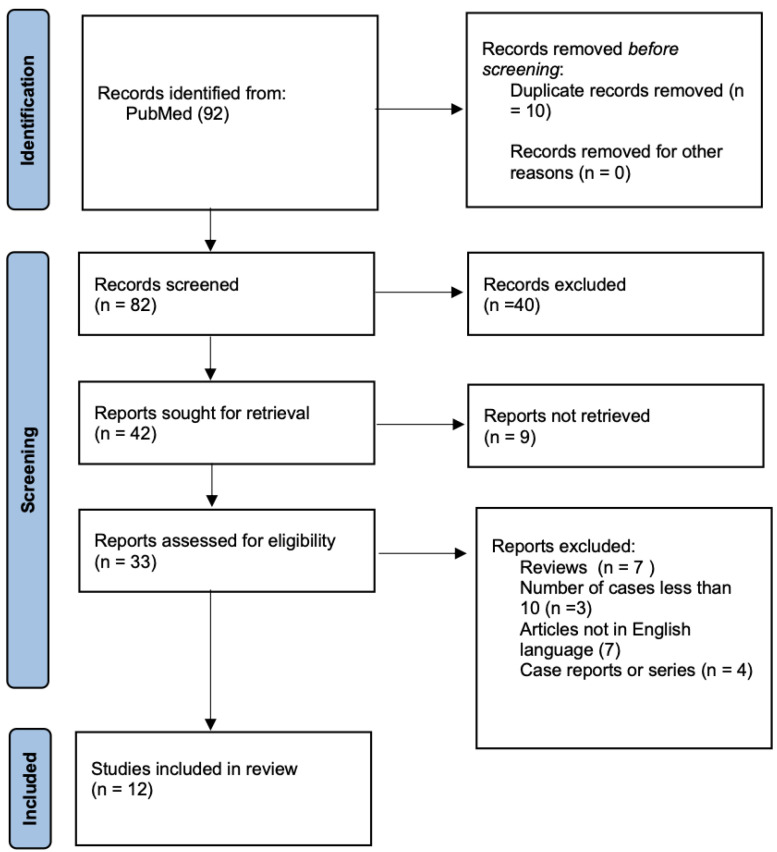
PRISMA flow-chart.

A systematic review was conducted in accordance with the Preferred Reporting Items for Systematic Reviews and Meta-Analyses (PRISMA) guidelines [11]. The outcomes of the study selection and the literature search are presented in the flow diagram in Figure 1. The search was performed from June 2024 to September 2024 using PubMed as the primary database. The strategy was restricted to articles published in English, with no limitations on publication date. The database was screened for search terms appearing in either the titles or abstracts. “Laparoscopic lateral suspension, “lateral suspension,” and “pelvic organ prolapse” were utilized for the research. These terms were searched using Boolean operators (AND, OR) to refine and optimize the query. Manual filters were applied to exclude duplicates and irrelevant records.

We included prospective cohort studies, randomized trials, or case series with more than 10 patients. Reviews, duplicate publications, non-relevant reports, abstracts, retrospective studies, and articles not published in English were excluded. Additionally, we excluded case reports or small case series, articles without full-text availability, and studies deemed irrelevant to our topic. The outcomes considered were success rate, recurrence rate, and re-operation rate. Study selection was independently performed by three authors (A.M., G.B.C.); two reviewers (A.M and G.B.C) independently assessed the risk of bias. In case of disagreement, a third reviewer (S.M.) was consulted. Titles and abstracts were screened for eligibility based on the inclusion and exclusion criteria, and any titles or abstracts deemed ineligible were excluded. Data were collected using an Excel spreadsheet.

Given the nature of the included non-randomized studies, the ROBINS-I (Risk Of Bias In Non-randomized Studies of Interventions) tool was applied. The majority of studies were judged to have a moderate risk of bias, primarily due to limitations in reporting confounding variables, lack of blinding, and incomplete follow-up data. A few studies presented serious risk of bias, particularly in the domains of outcome measurement and the selection of participants. Despite these limitations, all studies provided relevant clinical outcome data.

For each study, information was extracted on the following variables: year of publication, study type, patient age, menopausal status, history of previous POP surgery or hysterectomy, operative time, preoperative and postoperative POP stage (assessed and quantified using the Baden–Walker prolapse classification system or the POP-Q system), follow-up duration, success rate, recurrence rate, re-operation rate, subjective cure rate, and intraoperative and postoperative complications.

## 3. Results

The initial literature search yielded a total of 92 studies. After removing 10 duplicates, 82 unique articles were screened by reviewing their titles and abstracts to identify those meeting the inclusion criteria. Of the 82 studies screened, only 33 were assessed for eligibility. Based on our inclusion and exclusion criteria, twelve studies were ultimately included: nine prospective cohort studies, one prospective non-inferiority trial, and two prospective randomized trials.

The characteristics of the studies (year and study design) and baseline patient characteristics (age and history of prior pelvic reconstructive surgery) are summarized in Table S1. The preoperative and postoperative POP-Q stage (assessed by the same gynecologist), the follow-up duration, objective and subjective success rates, surgical failures, and re-operation rates are summarized in Table S2. All included studies were published between 2000 and 2024 and had an observational design. The total number of patients across the studies evaluated in this review was 1261. Eleven studies [12,13,14,15,16,17,18,19,20,21,22] investigated the laparoscopic approach, while one study [23] evaluated the laparoscopic and robotic approach.

It should be acknowledged that the quality of evidence varies substantially between randomized and observational studies, with the latter inherently limited by potential biases that may affect the interpretation of the findings.

The global median anatomical success rate (POP Q value ≤ 1) for the anterior compartment was 91.15%, ranging from 73% to 100%. For the central compartment, the median value was 94.95%, ranging from 78% to 100%. For the posterior compartment, the median value was 86.55% ranging from 50% to 100%. Subjective cure rates reported across studies were similarly encouraging, ranging from 78.4% to 96.2%. These findings indicate high levels of patient satisfaction, suggesting that most patients experienced significant relief from POP symptoms post-surgery, despite variations in the definitions of success across studies.

In Russo et al. the authors reported the first prospective non-inferiority trial between abdominal sacral colpopexy (ASC) and abdominal lateral suspension (ALS).

At the 6- and 12-month follow-up, which included POP-Q evaluation and targeted questions on subjective outcomes, the apical prolapse correction rates reached 92% for ALS and 94% for ASC. The anatomic objective cure was considered as POP-Q ≤ 1. Recurrence rates were 8% and 6%, respectively, and the *p*-value for non-inferiority was <0.01. Therefore, at the 12-month follow-up, this prospective multicenter trial demonstrates that ALS and ASC are equally effective in treating advanced apical prolapse with a significant *p*-value for non-inferiority < 0.01. The subjective cure rate (it was thought to be the postoperative absence of vaginal bulging) was higher than 90% for both procedures (95% ALS vs. 97% ASC, *p* = 0.655) [23].

In the first randomized prospective study reported in the literature, Dogan et al. compared the laparoscopic lateral suspension (with uterine preservation) to conventional sacrohysteropexy. When analyzing the anterior compartment, the laparoscopic sacrouteropexy group did not demonstrate significant variation between baseline and postoperative stages (*p* = 0.130). On the other hand, a notable difference was identified in the laparoscopic lateral suspension group, possibly due to the flexibility of the mesh in the prevesical region. On the other hand, both groups’ preoperative and postoperative apical staging differed significantly (*p* < 0.001, *p* < 0.001). Postoperative PQOL did not significantly differ between the lateral suspension and laparoscopic sacrouteropexy groups (*p* = 0.497) [21].

In the second randomized trial, Malanowska et al. compared laparoscopic sacrocolpopexy (LSC) and laparoscopic lateral suspension (LLS). In this study, objective success for apical prolapse was considered as achieving a POP-Q stage below II. All participants underwent laparoscopic supracervical hysterectomy. A total of ninety-three patients were evaluated, with four lost during follow-up. The anatomical success rates for laparoscopic sacrocolpopexy (LSC) reached 81.82% in the apical compartment and 95.22% in the anterior compartment. For laparoscopic lateral suspension (LLS), the success rates were 90% for apical support and 92.30% for anterior support. The difference observed in the anterior compartment between the two techniques (95.22% vs. 92.30%) did not reach statistical significance (*p* < 0.005). However, both groups’ apical prolapses showed a clinically and statistically significant anatomic improvement [22].

The mean operation time, the intraoperative and postoperative complications, the overall complication rate, and the mesh-related complications are summarized in Table S3. The range in operative time was 71.13 (±18.70) [18] to 254 ± 45 min [12]. In the study by Dogan et al., a significant difference was observed in the average duration of surgery between the groups, with the lateral suspension group reporting a mean operative time of 101.3 ± 10.3 min compared to 118.6 ± 10.9 min in the sacrouteropexy group (*p* < 0.001) [21]. Similarly, in Russo et al. the difference between the operating time was considered statistically significant (123 ± 33 min and 193 ± 55.6 min for ALS and ASC, respectively, *p*-value < 0.001) [23].

Following laparoscopic lateral suspension surgery, ten patients experienced bladder damage as one of the intraoperative complications [23].

There was one case, in Yassa et al., of conversion to laparotomy due to a postoperative complication grade IV according to the Clavien–Dindo classification [17], and there were another three cases of conversion to laparotomy in Malanowska et al.—in particular, one patient for the LLS, and two patients for LSC [22]. There were no cases of bowel injury. Across all patients analyzed in this systematic review, postoperative surgical complications were recorded as follows: 33 cases (2.6%) of Clavien–Dindo grade I; 38 cases (3.0%) of grade II; 9 cases (0.7%) of grade IIIa; and 8 cases (0.6%) of grade IIIb. Additionally, there was a single postoperative event classified as grade IV, which was associated with a false suspicion of CO_2_ embolism [18]. There were neither grade IV nor grade V complications. Problems relating to the mesh were managed separately.

Regarding Clavien–Dindo grade IIIa and IIIb complications, one patient experienced postoperative pain that was resolved by removing the lateral mesh fixation suture a few days after the procedure [14]. Additionally, a case of ilioinguinal neuralgia was managed promptly under local anesthesia by reopening the skin incision and cutting the suture positioned near the anterior superior iliac spine [13]. An automated defibrillator had to be implanted for a woman who was experiencing atrial fibrillation [18]. Re-operation was necessary for four trocar-related complications, including a hernia and a subcutaneous granuloma [16].

In the largest series of Veit and Rubin, on day 16, a patient with a ureterovaginal fistula—likely caused by the supracervical hysterectomy—had ureteral reimplantation, while a patient with a vaginal hematoma received surgical drainage [15].

Additionally, one case of postoperative melena and a subfascial hematoma requiring blood transfusion were identified, both categorized as Clavien–Dindo grade II complications [14]. Another patient developed an abdominal wall hernia at the site of a 12 mm trocar, classified as grade IIIa on the Clavien–Dindo scale [13]. Furthermore, two instances of mesh exposure were reported, with one assessed as grade II and the other as grade IIIa according to the Clavien–Dindo classification [24]. We report an overall rate of complications that ranges from 0 to 31.5%, with a median value of 7.2%.

In total, there were 34 (2.6%) cases of mesh erosion. Mesh erosion rates generally varied between 0% and 5.5% among papers, most likely as a result of differences in sample size, duration of the follow-up, type of prosthetic material, and kind of concurrent surgical procedures.

## 4. Discussion

Treating advanced apical prolapse remains a challenging clinical scenario. Sacral suspension through minimally invasive methods continues to be the gold standard for correcting multi-compartment and advanced apical prolapse. Despite being the most effective treatment for apical pelvic organ prolapse (POP), laparoscopic sacral colpopexy (LSCP) is highly invasive and carries the risk of rare but potentially severe complications associated with dissection of the sacral promontory [6]. Critical structures surrounding the presacral area—including the right ureter, left common iliac vein, and middle sacral arteries—contribute to a 1.0% risk of ureteral injury and a 4.4% risk of vascular injury, which may lead to blood loss and, in rare cases, death [24]. Consequently, innovative abdominal surgical techniques, such as laparoscopic lateral suspension (LLS), have been developed in recent years to treat multi-compartment and advanced apical POP [25]. These techniques aim to provide tailored care and better patient outcomes by avoiding sacral dissection, reducing intraoperative complications, shortening the learning curve, and offering comparable effectiveness in treating apical prolapse [25]. ALS presents a more practical alternative to sacrocolpopexy for managing apical or mixed anterior and apical prolapse [26]. Nevertheless, current evidence on laparoscopic lateral suspension following the Dubuisson technique remains limited and partly conflicting, especially concerning the definitions of success, choice of materials, and long-term outcomes. In terms of overall success rates, our study found a median value of 91.15% for the anterior compartment, 94.95% for the central compartment, and 86.55% for the posterior compartment. Several studies indicate that only a small proportion of women experience posterior prolapse grades above II, suggesting that LLS may not be suitable for severe posterior prolapse. In cases of advanced prolapse involving the posterior and apical compartments, reinforcing the recto-vaginal space with mesh during sacrocolpopexy—by positioning the mesh posteriorly—significantly improves support of the posterior vaginal wall, establishing sacrocolpopexy as the preferred surgical approach for complex, multi-compartment prolapse [27]. The favorable results observed in the anterior compartment with the LLS technique may be attributed to the deep anterior dissection in the prevesical space, which is a hallmark of the LLS approach. LLS is also safe and feasible, even for obese patients [28]. In their systematic review encompassing 1066 laparoscopic lateral suspension procedures, Campagna et al. documented anatomical success rates exceeding 90% for apical support and over 88% for the anterior compartment [10]. Regarding subjective cure rates, the majority of studies report favorable patient satisfaction, often exceeding 90%. However, recurrence rates vary, underscoring the importance of patient-reported outcomes.

In randomized studies comparing the two techniques—colposacropexy (the current gold standard) and laparoscopic lateral suspension—the results suggest that LLS is not inferior to sacrocolpopexy in terms of both anatomical and subjective outcomes. The superiority of lateral suspension in reducing bladder descent was demonstrated by objective measurements. The comparison of these techniques is challenging due to variations in the definition of successful procedures, follow-up durations, and preoperative prolapse severity. Our findings emphasize the need for standardized metrics to evaluate surgical efficacy.

Surgical time, a critical factor influencing recovery, varied significantly, ranging from 71.13 ± 18.70 min to 254 ± 45 min. This variability is largely attributable to concurrent procedures, such as hysterectomies and incontinence treatments, often performed alongside prolapse repairs. Data in Table S2 illustrate intraoperative and postoperative complications, showing a manageable complication rate, though mesh-related issues require careful consideration. Furthermore, the complications associated with LLS, as detailed in our findings, demonstrated a low incidence of severe postoperative complications, which is promising. Notably, the mesh erosion rate varied significantly across studies, indicating that factors such as sample size, type of mesh, and surgical technique may influence these outcomes.

Uterine preservation is another crucial consideration in counseling patients with POP, according to recent scientific evidence. Several meta-analyses have explored the issue of uterine preservation in prolapse correction treatments. LLS is recommended for women who wish to preserve their uterus and maintain an active sexual life [10]. Veit et al. reported superior anatomical results in the anterior compartment with the uterine-sparing approach in LLS. A recent meta-analysis by Tius et al. highlighted that laparoscopic sacrocolpopexy with concurrent hysterectomy—whether total or subtotal—demonstrates superior anatomical and subjective success compared to sacrohysteropexy alone [25]. Importantly, it does not appear to carry a higher risk of mesh exposure. The two techniques show no significant differences in recurrence rates or re-operation rates, and laparoscopic sacrohysteropexy could be an alternative for patients seeking to preserve the uterus due to concerns about body image, femininity, or fertility [25]. Although the approach offers a minimally invasive alternative with potential benefits in uterine preservation, the lack of robust randomized trials still hampers definitive conclusions. This review has some limitations. Only articles published in English were included, and no attempts were made to contact study authors for missing data. Additionally, a meta-analysis was not feasible due to clinical and methodological heterogeneity among included studies. Future studies should focus on standardized reporting and extended follow-up. Additionally, artificial intelligence may play a role in advancing this field, supporting preoperative planning and enabling more personalized treatment strategies through predictive analytics.

## 5. Conclusions

This systematic review underscores the efficacy and safety of laparoscopic lateral suspension (LLS) for the treatment of pelvic organ prolapse (POP), demonstrating favorable anatomical and functional outcomes. The results indicate that while LLS provides substantial benefits, careful patient selection and thorough preoperative evaluation are essential for optimizing results. Ongoing follow-up is critical to assess long-term effectiveness and manage potential complications, particularly those associated with mesh use. Further research is needed to refine surgical techniques and deepen our understanding of the long-term effects of LLS across diverse patient populations.

## Supplementary Materials

The following supporting information can be downloaded at https://www.mdpi.com/article/10.3390/jcm14093056/s1, Table S1: Patients’ baseline characteristics. Table S2: Results. Table S3: Intraoperative and postoperative complications.

## Abbreviations

The following abbreviations are used in this manuscript:

POP	Pelvic organ prolapse
LLS	Laparoscopic lateral suspension
LSC	Laparoscopic sacrocolpopexy
CD	Clavien–Dindo
ASC	Abdominal sacral colpopexy
ALS	Abdominal lateral Suspension
