# Supplementary material for: Laparoscopic Lateral Suspension (LLS) for Pelvic Organ Prolapse (POP): Update and Systematic Review of Prospective and Randomised Trials
Source: J Clin Med. 2025 Apr 29;14(9):3056. doi: 10.3390/jcm14093056 (PMC12072532; doi:10.3390/jcm14093056)
Supplement: Supplementary file 1 [file jcm-14-03056-s001.zip › TABLE S1 .pdf]

STUDY	TYPE OF STUDY	N.	AGE Mean /median	post menopausal n (%)	previous hysterectomy n (%)	previous POP surgery n (%)
Dubuisson et al. (2000)	Prospective	35	51.1 ± 13.3	15	0	2(5.7)
Dubuisson et al. (2008)	Prospective	73	55 (36–81)	49 (67.1)	22 (30.1)	17 (23.3)
Dubuisson et al. (2013)	Prospective	73	63 (39–83)	/	73 (100)	31 (42.5)
Veit-Rubin et al. (2017)	Prospective	417	57.7 ± 11.5	292 (71.1)	74 (17.8)	69 (16,5)
Malowska et al (2019)	Prospective	64	59.4 ± 9.3	60 (93.7)	0	0

Yassa M et al. (2019)	Prospective	17	52.3 ± 11.7	9 (53)	0	2 (18)
Gil Ugarteburu et al. (2019)	Prospective	20	69.2 ± 9.1	0	4 (20)	5 (25)
Chatziioannidou K et al (2021)	Prospective	88	59.6 ± 11.1	56 (70.9%)	7 (8.9)	10 (12.7%
Aksin et al. (2023)	Prospective	41	51.45 ± 11.51	/	0	/
Russo et al (2023)	Prospective non inferiority trial (ALS vs ASC)	200 | 100	63.1 ± 8.1| 64.9 ± 8.8	169 (84.5) | 92 (92)	13 (6.5) | 67(67)	/

Dogan et al (2024)	Prospective randomized LLS vs LSC/sacrohysteropexy	22 | 22	51.2 ± 8.4 | 52.8 ±8.6	11 (50) | 10 (45.5)	0 | 0	0 | 0
Malanowska-Jarema et al. (2024)	Prospective randomized LLS vs LSC/sacrocolpopexy	46 | 43	59.49 ±8.84 | 58.14 ±8.28	42 (93.02) | 41 (95.35)	46 |43	0|0

LEGEND:

ALS abdominal lateral suspensions
ASC abdominal sacral colpopexy
LLS laparoscopic lateral suspension
LSC laparoscopic sacrocolpopexy
