# Supplementary material for: Laparoscopic Lateral Suspension (LLS) for Pelvic Organ Prolapse (POP): Update and Systematic Review of Prospective and Randomised Trials
Source: J Clin Med. 2025 Apr 29;14(9):3056. doi: 10.3390/jcm14093056 (PMC12072532; doi:10.3390/jcm14093056)
Supplement: Supplementary file 1 [file jcm-14-03056-s001.zip › TABLE S2.pdf]

STUDY	Preoperative POP stage N (%)			Postoperative POP stage N (%)			FOLLOW UP, MONTHS (N)	SUCCESS RATE (%)	RECURRENCE RATE (%)	REOPERATION RATE (%)	SUBJECTIVE CURE RATE (%)
	Anterior	Apical	Posterior	Anterior	Apical	Posterior					
Dubuisson et al. (2000)	II 18 (51.4) III 16 (45.7)	II 20 (57.1) III 11(31)	II 15 (42.9) III 2 (5.7)	0 35(100)	0 35(100)	0 28(80) I 7(20)	5 (±4.6)	Anterior 100 Apical 100 Posterior 80	Overall 20 Anterior 0 Apical 0 Posterior 20	0	90.9
Dubuisson et al. (2008)	0 5(6.8) I 1(1.4) II 36 (49.3) III 30 (41.1) IV 1 (1.4)	0 6 (8.2) I 23 (31.5) II 37 (50.7) III 5 (6.8) IV 2(2.7)	0 12(16.4) I 28(38.3) II 27 (36.9) III 6(8.2)	0 66 (90.4) I 4(5.5) II 2(2.7) III 1(1.4)	0 64(87.7) I 5(6.8) II 3(4.1) III 1(1.4)	0 63 (86.3) I 7 (9.6) II 3(4.1)	19 (12–41)	Overall 87.7 Anterior 95,9 Apical 94,6 Posterior 97,3	Overall 12.3 Anterior 4.1 Apical 5.5 Posterior 2.7	6.8	100
Dubuisson et al. (2013)	0 7(9.6) I 2(2.7) II 28 (38.3) III 35 (47.9) IV 1(1.4)	I 21 (28.8) II 24 (32.9) III 27 (37) IV 1(1.4)	0 12(16.4) I 13(17.8) II 35 (47.9) III13 (17.8)	0 62(84.9) I 9(12.3) II 2(2.7)	0 66(90.4) I 3(4.1) II 3(4.1) III 1(1.4)	0 58(79.5) I 6(8.2) II 9 (12.3)	17.5 (1–67)	Overall 82,7 Anterior 97,3 Apical 98,6 Posterior 87,7	Overall 17.8 Anterior 2.7 Apical 1.4 Posterior 12,3	8.2	n/a
Veit-Rubin et al. (2017)	Ba ≥ -1 39 (95.4)	C ≥ -1 384 (92.1)	Bp≥ -1 375 (89.9)	n/a	n/a	n/a	12	Anterior 90.6 Apical 92.6	Overall n/a Anterior 9.4 Apical 7.4 Posterior 15	7.3	78.4

								Posterior<br>85			
Malowska et al (2019)	Ba<br>1.67 ±<br>1,13	C<br>0.06 ±<br>1.63	Bp 0.47 ±<br>1,01	Ba - 1.63<br>± 1,11	C -5.55 ±<br>2,53	Bp -<br>2.19 ±<br>2,29	12	Anterior<br>76.2.<br>Apical<br>84.4	Overall 12.5<br>Anterior 3.1<br>Apical 4.7<br>Posterior. 4,7	10.9	95.3
Yassa M et al. (2019)	0<br>1(5.9)<br>I<br>3(17.6<br>)<br>II 6<br>(35.3)<br>III 5<br>(29.4)<br>IV 2<br>(11.8)	II 2<br>(11.8)<br>III 6<br>(35.3)<br>IV 9<br>(52.9)	0<br>11(64.7)<br>I 5(29.4)<br>II 1(5.9)	0<br>11(64.7)<br>I 4 (23.5)<br>II 2<br>(11.8)	0 17(100)	0 12<br>(70.6)<br>I 4<br>(23.5)<br>II 1<br>(5.9)	23.93<br>(15.3– 28.8)	Anterior<br>88,2<br>Apical<br>100	Overall 11.8	5,9	94.12
Gil Ugarteburu et al. (2019)	II 9<br>(45)<br>III<br>11(55)	0<br>7(35)<br>I<br>5(25)<br>II<br>7(35)<br>III<br>1(5)	0 10(50)<br>I 7(35)<br>II 3(15)	0 9(45)<br>I 5(25)<br>II 5(25)<br>III 1(5)	0 17(85)<br>I 3(15)	0 13(65)<br>I 6(30<br>II 1(5)	12.8 (±5.7)	Anterior<br>90<br>Apical<br>100	Overall 10	0	90
Chatziioannido u K et al (2021)	I 3<br>(3.8)<br>II 16<br>(20.3)<br>III 57<br>(72.2)<br>IV<br>3(3.8)	0 5<br>(6.3)<br>I 11<br>(13.9)<br>II 32<br>(40.5)<br>III 27<br>(34.2)<br>IV 4	0 13<br>(16.5)<br>I 25<br>(31.6)<br>II 38<br>(48.1)<br>III 3 (3.8)	0 24<br>(30.4)<br>I 27<br>(34.2)<br>II 28<br>(35.4)	0 57<br>(72.2)<br>I 14<br>(17.7)<br>II 6 (7.6)<br>III 2 (2.5)	0 36<br>(45.6)<br>I 18<br>(22.8)<br>II 24<br>(30.4)<br>III 1<br>(1.3)	3.4	Overall<br>87.3	7.6	5.1	96.2

		(5.1)									
Aksin et al. (2023)	Ba 2.90 ± 1.42	C 3.81 ± 2.91	Bp 1.03 ± 2.79	Ba−1.48 ± 1.93	C −5.52 ± 3.90	Bp −0.96 ± 2.84	12	Anterior 73 Apical 78	Anterior 29.3 Apical 22,3	0	78.1
Russo et al (2023)	ALS II 39 (19,5) III 94 (47) IV 53 (26.5) ASC II 15 (15) III 35 (35) IV 22 (22)	ALS II 54 (27) III 101 (50.5) IV 45 (22.5) ASC II15 (15) III 50 (50) IV 35 (35)	ALS II 10 (5%) III 2 (1 %) IV 0 (0%) ASC II 24 (24) III 20 (20) IV 11 (11)	n/a	n/a	n/a	6, 12	ALS Anterior 83.9 Apical 92 Posterior 50 ASC Anterior 80.6 Apical 94 Posterior 85.4	ALS Anterior 16.1 Apical 8 Posterior 50 ASC Anterior 19.4 Apical 6 Posterior 14.6	ALS Anterior 2.1 Apical 2.5 Posterior 8.3 ASC Anterior 5.5 Apical 2 Posterior 0	95 | 97

Dogan et al (2024)	LLS<br>I 5<br>(22.7)<br>II 16<br>(72.7)<br>III 1<br>(4.5)<br>IV<br>0(0)<br><br>LSC<br>I 3<br>(13.6)<br>II 18<br>(81.8)<br>III 1<br>(4.5)<br>IV<br>0(0)	LLS<br>I 0 (0)<br>II 4<br>(18.2)<br>III 8<br>(36.4)<br>IV<br>10<br>(45.5)<br><br>LSC<br>I 0(0)<br>II 5<br>(22.7)<br>III 6<br>(27.3)<br>IV 11<br>(50)	LLS<br>I 8<br>(36.4)<br>II 9<br>(40.4)<br>III 0(0)<br>IV 0(0)<br><br>LSC<br>I 9<br>(40.9)<br>II 10<br>(45.5)<br>III 0 (0)<br>IV 0(0)	LLS<br>I 7 (31.8)<br>II 0 (0)<br>III 0 (0)<br>IV 0 (0)<br><br>LSC<br>I 5 (22.7)<br>II 15<br>(68.2)<br>III 1<br>(4.5)<br>IV 0(0)	LLS<br>I 1 (4.5)<br>II 0 (0)<br>III 0 (0)<br>IV 0 (0)<br><br>LSC<br>I 1 (4.5)<br>II 0 (0)<br>III 0 (0)<br>IV 0(0)	LLS<br>I 9<br>(40.9)<br>II 6<br>(27.3)<br>III 0 (0)<br>IV 0 (0)<br><br>LSC<br>I 9<br>(40.9)<br>II 9<br>(40.9)<br>III 0 (0)<br>IV O (0)	12	LLS<br>Anterior<br>100<br>Apical 100<br>Posterior<br>100<br><br>LSC<br>Anterior<br>95<br>Apical 100<br>Posterior<br>100	0 | 0	0<br>| Anterior 4.5	n/a
Malanowska-Jarema et al. (2024)	LLS<br>II 9<br>(19,5)<br>III 17<br>(36,9)<br><br>LSC<br>II 5<br>(11,6)<br>III 16<br>(37)	LLS<br>II<br>26<br>(56,5)<br>III 20<br>(43,5)<br><br>LSC<br>II 21<br>(49)<br>III<br>22<br>(51)	n/a	LLS<br>I 22<br>(47,8)<br>II 3 (6,5)<br>III 1<br>(2,17)<br><br>LSC<br>I 17 (39)<br>II 3 (6.9)<br>III 1 (2,3)	LLS<br>I 41 (89)<br>II 1 (2,17)<br>III 4 (8,6)<br><br>LSC<br>I 39<br>(90,7)<br>III 4<br>(9.3)	n/a	12	LLS<br>Anterior<br>92.3<br>Apical 90<br><br>LSC<br>Anterior<br>95.22<br>Apical<br>81.82	LLS<br>Anterior 8.6<br>Apical 10<br>LSC<br>Anterior 9.3<br>Apical 9.3	n/a	n/a

LEGEND:

n/a : not available
