# Supplementary material for: Laparoscopic Lateral Suspension (LLS) for Pelvic Organ Prolapse (POP): Update and Systematic Review of Prospective and Randomised Trials
Source: J Clin Med. 2025 Apr 29;14(9):3056. doi: 10.3390/jcm14093056 (PMC12072532; doi:10.3390/jcm14093056)
Supplement: Supplementary file 1 [file jcm-14-03056-s001.zip › TABLE S3 .pdf]

INTRAOPERATIVE COMPLICATIONS						POSTOPERATIVE COMPLICATIONS ACCORDING TO CLAVIEN DINDO CLASSIFICATION								MESH RELATED COMPLICATION N (%)
Study	Patients	Median/mean OT (min)	BLADDER INJURY N (%)	BOWEL INJURY N (%)	CONVERSION TO LPT N (%)	1N (%)	2N (%)	3aN (%)	3bN (%)	4aN(%)	4bN (%)	5N (%)	Overall complication rate %	
Dubuisson et al. 2000	35	254(±45)	1 (2.9)	0 (0)	0 (0)	2 (5.7)	2 (5.7)	1 (2.9)	0 (0)	0 (0)	0 (0)	0 (0)	20	1 (2.9)
Dubuisson et al. 2008	73	208 (196– 220)	0 (0)	0 (0)	0 (0)	4 (5,5)	13 (17,8)	1 (1.4)	1 (1.4)	0 (0)	0 (0)	0 (0)	31,5	4 (5.5)
Dubuisson et al. 2013	73	193 (90–300)	1(1.3)	0 (0)	0 (0)	1 (1,3)	2 (2,7)	0 (0)	0 (0)	0 (0)	0 (0)	0 (0)	5,4	4 (5.5)
Veit-Rubin et al. 2017	417	201 (±52.3)	4 (0.9)	0 (0)	0 (0)	10 (2.4)	19 (4.6)	0 (0)	5 (1,2)	0 (0)	0 (0)	0 (0)	9	17 (4,3)
Malanoska et al. 2019	64	not clear	2 (3.1)	0 (0)	0 (0)	2 (3.1)	0 (0)	0 (0)	0 (0)	0 (0)	0 (0)	0 (0)	6,2	0 (0)
Yassa M. et al. 2019	17	108.8 (±29.8)	0 (0)	0 (0)	1(5.9)	0 (0)	0 (0)	0 (0)	0 (0)	1 (5.9)	0 (0)	0 (0)	11,8	0 (0)

<table>
<tr><td>Gil Ugarteburu et al. 2019</td><td>20</td><td>78.4 (±29.7)</td><td>0 (0)</td><td>0 (0)</td><td>0 (0)</td><td>2 (10)</td><td>0 (0)</td><td>0 (0)</td><td>1(5)</td><td>0 (0)</td><td>0 (0)</td><td>0 (0)</td><td>15</td><td>0 (0)</td></tr>
<tr><td>Chatziioannidou K et al 2021</td><td>88</td><td>180.0 (180–240)</td><td>0 (0)</td><td>0 (0)</td><td>0 (0)</td><td>0 (0)</td><td>0 (0)</td><td>0 (0)</td><td>0 (0)</td><td>0 (0)</td><td>0 (0)</td><td>0 (0)</td><td>0</td><td>0 (0)</td></tr>
<tr><td>Aksin et all 2023</td><td>41</td><td>71.13 (± 18.70)</td><td>0 (0)</td><td>0 (0)</td><td>0 (0)</td><td>0 (0)</td><td>0 (0)</td><td>0 (0)</td><td>0 (0)</td><td>0 (0)</td><td>0 (0)</td><td>0 (0)</td><td>0</td><td>4 (9.9)</td></tr>
<tr><td>Russo et al 2023</td><td>200 | 100</td><td>123 ± 33 | 193 ± 55.6</td><td>0 (0) | NOT CLEAR</td><td>0 (0) | not clear</td><td>0 (0) | 0(0)</td><td>12 (6.0) | 0 (0)</td><td>0 (0) | 2(2.0)</td><td>4 (2.0) | 3 (3.0)</td><td>0 (0) |0(0)</td><td>0 (0) |0(0)</td><td>0 (0) | 0(0)</td><td>0 (0) | 0(0)</td><td>8 | 6</td><td>2 (1.0) | 2 (2.0)</td></tr>
<tr><td>Dogan et al 2024</td><td>22 | 22</td><td>LLS
101.3 ± 10.3
LSC
118.6 ±10.9</td><td>0 (0) | 0 (0)</td><td>0 (0) | 0(0)</td><td>0 (0) | 0(0)</td><td>n/a</td><td>n/a</td><td>n/a</td><td>n/a</td><td>n/a</td><td>n/a</td><td>n/a</td><td>4.5|9.1</td><td>n/a</td></tr>
<tr><td>Malanowska-Jarema et al.2024</td><td>46| 43</td><td>LLS
160.3 ± 43.91
LSC
168.26 ± 37.37</td><td>2 (4,3) | 0(0)</td><td>0 (0) | 0(0)</td><td>1 (2.17) | 2 (4,6)</td><td>0(0) | 0(0)</td><td>0(0)| 0 (0)</td><td>0(0) | 0(0)</td><td>0(0) | 1 (2,3)</td><td>0 (0) | 0(0)</td><td>0 (0) |0 (0)</td><td>0 (0) | 0 (0)</td><td>6,4|6,9</td><td>0 (0) | 0(0)</td></tr>
</table>

LEGEND:

n/a : not available
